# Early bone formation around immediately placed two-piece tissue-level zirconia implants with a modified surface: an experimental study in the miniature pig mandible

**DOI:** 10.1186/s40729-022-00437-z

**Published:** 2022-09-14

**Authors:** Roland Glauser, Peter Schupbach

**Affiliations:** 1 Cosmodent Clinic Zürich, Zürich, Switzerland; 2 Schupbach Ltd, Histology and Electron Microscopy, Thalwil, Switzerland

**Keywords:** Animal experiment, Miniature pig, Zirconia implant, Osseointegration, Immediate implant, Transmucosal implant, Bone-to-implant contact, Histology

## Abstract

**Purpose:**

To histologically examine early bone formation around transmucosal implants and to evaluate the influence of surface characteristics on early peri-implant bone healing using a miniature pig model. For this, commercially available dental implants with a rough zirconia (YTZP) surface were compared to surface-modified Ti control implants at 4 and 8 weeks after placement.

**Methods:**

Immediately following the extraction of six mandibular premolars, 20 two-piece, tissue-level, screw-shaped YTZP implants (Patent™ Standard Zirconia Implant ø4.1 × 11 mm) with a modified rough blasted before sintering surface were inserted in four adult miniature pigs. In addition, four titanium (Ti) tissue-level implants (Straumann^®^ Standard RN ø4.1 × 10 mm Roxolid^®^) with a moderate surface (SLActive^®^), one per animal, were placed as control implants. A histological analysis was performed on the hard tissues after 4 and 8 weeks of transmucosal healing.

**Results:**

The results show a high rate of osseointegration of the test YTZP dental implants at 4 and 8 weeks following insertion. At 4 weeks, a bone-to-implant contact ratio (BIC) of 73.7% (SD ± 16.8) for the test implants (*n* = 10) and 58.5% for the first control implant was achieved. The second control implant had to be excluded from analysis. At 8 weeks, a BIC of 82.4% (SD ± 16.9) for the test implants (*n* = 9) and 93.6% (SD ± 9.1) (*n* = 2) for the control implant was achieved. No statistical difference was observed comparing 4 and 8 weeks YTZP data (*p* = 0.126).

**Conclusions:**

The results indicate a predictable osseointegration of immediate zirconia implants with a modified YTZP implant surface and a high degree of BIC present at 4 weeks following insertion. After 8 weeks of healing both the zirconia implants and the Ti implants show a BIC indicating full osseointegration. Further studies involving a larger sample size with more time points are needed to confirm these results.

## Background

The long-term clinical success of dental implants, in terms of function and esthetics, relies on sustained tissue integration. Osseointegration, defined as direct bone apposition to the implant surface, can occur with implants made of various materials [1, 2, 3, 4, 5, 6, 7, 8, 9, 10]. A wide range of materials have been used for dental implants, with commercially pure (cp) titanium (Ti) as the most common. Surface modification of Ti implants by hydroxyapatite coating, sandblasting, and/or acid etching is usually performed to increase bone apposition [11]. Although cp Ti exhibits high biocompatibility and favorable mechanical properties, a disadvantage is the grayish color, which can lead to undesirable esthetics when mucosal tissue retracts and the Ti surface becomes exposed. In addition, the potential accumulation of Ti particles in local lymph nodes is a limitation [12, 13, 14, 15].

Initially, various ceramics were used as implant material; however, their use is currently insignificant [4, 16, 17, 18]. Yttria-stabilized zirconia (YTZP), a ceramic material with wide application and accepted long-term results in the field of orthopedic medical implants, has been introduced as a new dental implant material [19, 20, 21, 22, 23]. Its successful application was demonstrated in several preclinical and clinical studies as evidenced by excellent tissue integration and positive clinical outcomes [19, 20, 21, 22, 23, 24, 25, 26, 27].

Moreover, the inflammatory response induced by ceramic particles is considerably lower compared with that induced by Ti particles, clearly indicating the biocompatibility of such ceramics [28, 29].

However, there are few studies that have explored the healing mechanisms around zirconia implants, also referred to ZrO_2_ implants. Some studies indicate that smooth ZrO_2_ surfaces result in comparatively long healing periods, although these only included single time points without controls [19, 20, 23, 24].

The goal of this study was to qualitatively and quantitatively examine early bone apposition and the bone healing mechanisms of commercially available rough zirconia dental implants at 4 and 8 weeks after insertion and to compare the tissue reaction to surface-modified Ti control implants. The null hypothesis assumed was that the rough zirconia implant surfaces tested would perform similarly to well-documented reference titanium surfaces, with similar bone-to-implant contact (BIC) values as the primary outcome parameter and similar new bone formation as the secondary outcome parameter.

## Methods

### Study implants

**Fig. 1:**
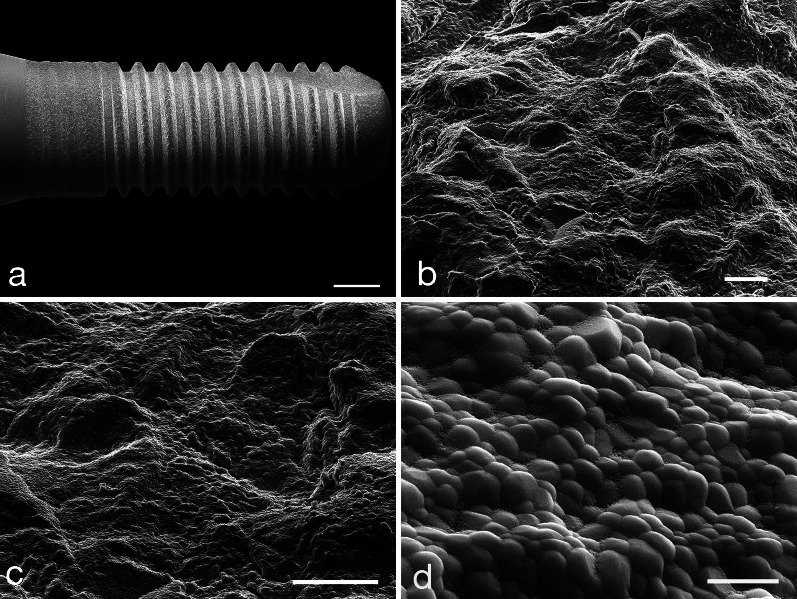
SEM micrographs of an YTZP implant. **a** Magnification: × 28; scale bar: 2 mm; **b** magnification: × 2500; scale bar: 0.01 mm; **c** magnification: × 5000; scale bar: 0.02 mm; **d** magnification: × 50,000; scale bar: 1 µm

Twenty commercially available two-piece screw-shaped tissue-level YTZP implants with a modified, roughened surface were tested (Patent™ Standard Zirconia Implant, 2-piece, REF 2S4111-2p ø4.1 × 11 mm EP 5.2, LOT 40290920c01; Zircon Medical Management AG) (Fig. 1a–d). The implants are produced from yttria-stabilized ZrO_2_ in a patented manufacturing process. The blasted before sintering (BBS) surface of the intraosseous portion of the YTZP implant has a surface roughness (R_a_) of 5.7 µm. The transmucosal portion has a machined surface with a surface roughness of 1.25 µm. Straumann Ti implants [Straumann^®^ Standard RN ø4.1 mm Roxolid^®^ (85% Ti, 15% ZrO_2_) SLActive^®^ 10 mm, REF 033.532 s, LOT EPW62, Straumann Group AG] with an intraosseous SLActive^®^ surface roughness of R_a_ = 2.2 µm and a transmucosal portion with machined surface served as control. Surface roughness data are given by the respective manufacturer.

### Specipig^®^ animal model

The study was performed at Specipig Barcelona, a certified and authorized breeding, supplier and animal experimentation center. Ethical approval for this study was provided by the Direcció General del Medi Natural i Biodiversitat, Servei de Biodiversitat i Protecció dels Animals (C/Dr. Roux, 80, 08017 Barcelona, document IMP-115).

Four miniature pigs (species *Sus scrofa domesticus* and Specipig^®^ miniature breed) were used for the study. The pigs were male, older than 20 months, and weighed more than 20 kg. They were identified by their ear tags. Antibiotics were administered on the day of surgery and continued for 1 week. Two pigs were randomly assigned to the 4-week, and the remaining to the 8-week healing group following surgery. The pigs were provided soft food for the first 4 weeks after surgery, after that, they were provided regular pellet-based food. Healing and well-being were monitored regularly.

### Surgical procedure

**Fig. 2:**
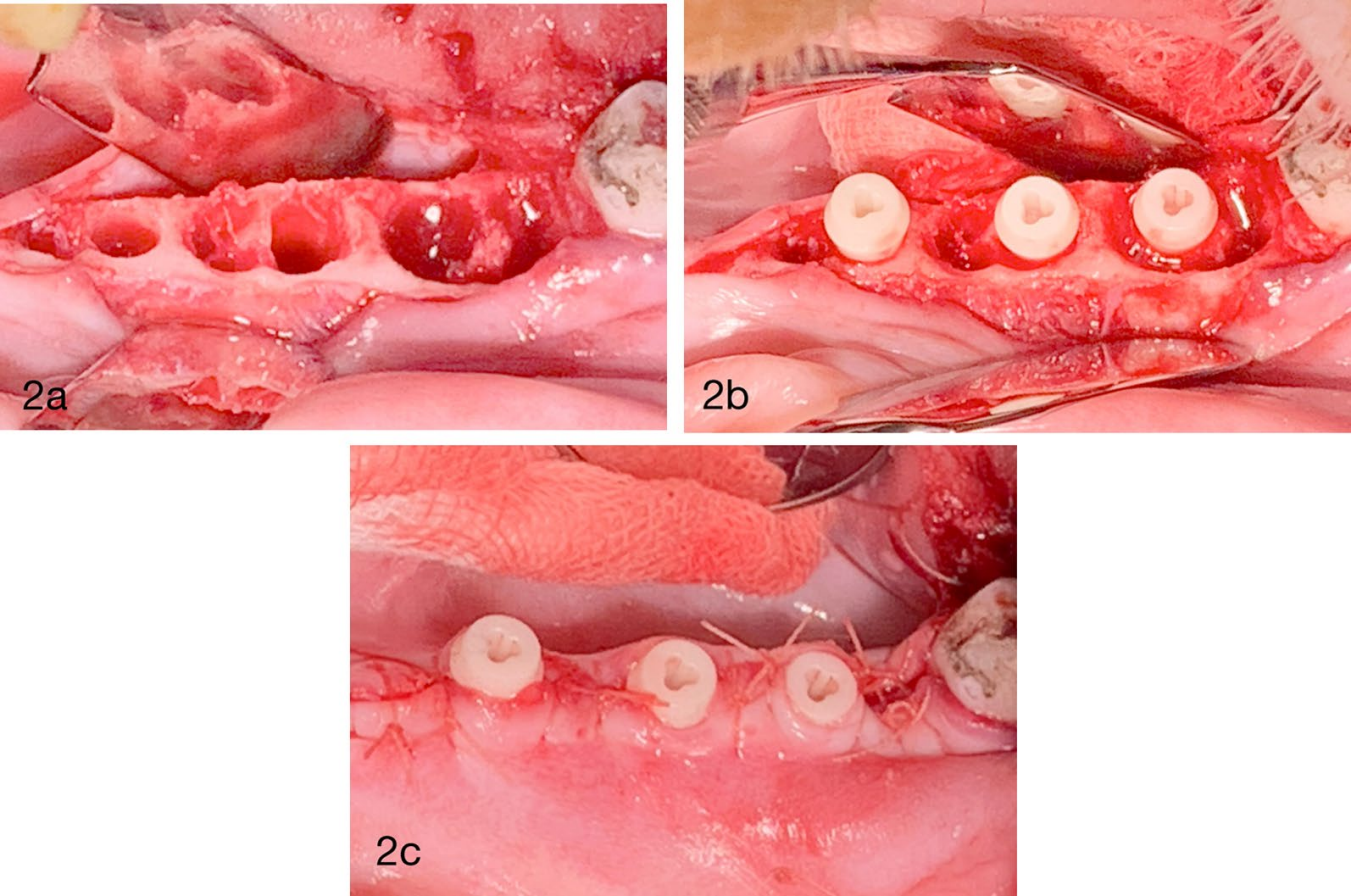
a. Occlusal view of post extraction morphology. b. Postoperative condition following implant insertion and flap adaptation

Under full sedation and local anesthesia, a flap was elevated in the mandibular premolar region to provide good visibility and access to the sites. All three premolars on each side were extracted without trauma (Fig. 2a). Because all removed teeth were twin-rooted, any remaining root fragments were carefully removed, and the extraction sockets were systematically curetted and rinsed using sterile saline solution in order to clean all sites properly. For each tooth position, implant site preparation was conducted in one of the sockets according to the manufacturer’s instructions, resulting in three osteotomies on each side of the mandible. Immediately after extraction, five test implants and one Ti control implant were randomly assigned and placed in each animal with an average insertion torque of 36 ± 9.8 Ncm. Neither surgical bone leveling nor socket grafting or membrane was applied before flap closure. Flaps were carefully adapted and closed with resorbable sutures (Vicryl 5-0 Ethicon, Johnson & Johnson). All test and control implants were subjected to transmucosal healing (Fig. 2b).

### Scanning electron microscopic (SEM) evaluation of the YTZP implant surface

Three test YTPZ implants were removed from the original package and mounted on alumina stub holders. The implants were sputter-coated with a 6-nm-thick platinum layer, and examined by scanning electron microscopy (Zeiss Supra 40VP; Zeiss, Oberkochen, Germany).

### Termination and preparation of ground sections for light microscopy

After 4 and 8 weeks of transmucosal healing, two animals at a time were killed and block sections were harvested.

The blocks were fixed by immersing in 10% neutral-buffered formalin and prepared for non-demineralized ground sections according to the technique of Donath and Breuner [30]. The specimens were washed with 0.01 M PBS buffer (Sigma-Aldrich) and dehydrated for approximately 4 days at each step in an ascending series of ethanol–pure water, with absolute ethanol (Sigma-Aldrich) for the final step.

The specimens were infiltrated with a graded series of ethanol and Technovit^®^ 7200 VLC (Kulzer, Wehrheim, Germany) embedding resin for at least 12 days at standard temperature with constant shaking. The specimens were then placed into three consecutive containers of 100% Technovit^®^ 7200 VLC for 24 h. Following dehydration and infiltration, the specimens were placed into embedding molds filled with fresh Technovit^®^ 7200 VLC and polymerized with 450-nm light for 10 h, while cooling with running tap water to avoid temperatures exceeding 40 °C.

Polymerized blocks were sliced in the bucco-lingual direction using an Exakt cutting unit (Exakt, Norderstedt, Germany). The slices were reduced by microgrinding and polishing with an Exakt grinding unit to an even thickness of 80–120 µm. A final polish was applied with 0.1-μm diamond polishing paste. The sections were stained with Sanderson’s RBS (Dorn & Hart, Villa Park, US) and counterstained with acid fuchsin. The sections were cover-slipped for analysis using a Leica M205A stereo light microscope and a Leica DM6B light microscope.

### Data analysis

The total percentage of bone-to-implant contact (BIC) as a primary outcome measurement was evaluated using ImageAccess software (Imagic, Glattbrugg, Switzerland). Both the lingual and buccal BIC per implant was measured beginning at the point of first BIC to the last point of BIC. The mean percentage of BIC and the standard deviation (SD) was calculated for test and control implants after 4 and 8 weeks.

Statistical analysis was performed using two-tailed *t*-tests for the comparison of the BIC values of the YTZP test implants 4 weeks versus 8 weeks following insertion. Crestal bone resorption was measured from the implant shoulder to the most coronal BIC and evaluated using a two-tailed *t*-test.

## Results

The healing was uneventful, and no implants were lost during the healing phase. At 4 weeks, 10 implants from the test group and two from the control group were available. One control group implant was accidentally placed in the root remnant of a tooth and caused severe inflammatory reactions and bone resorption. This implant was excluded from further histologic evaluation.

**Fig. 3:**
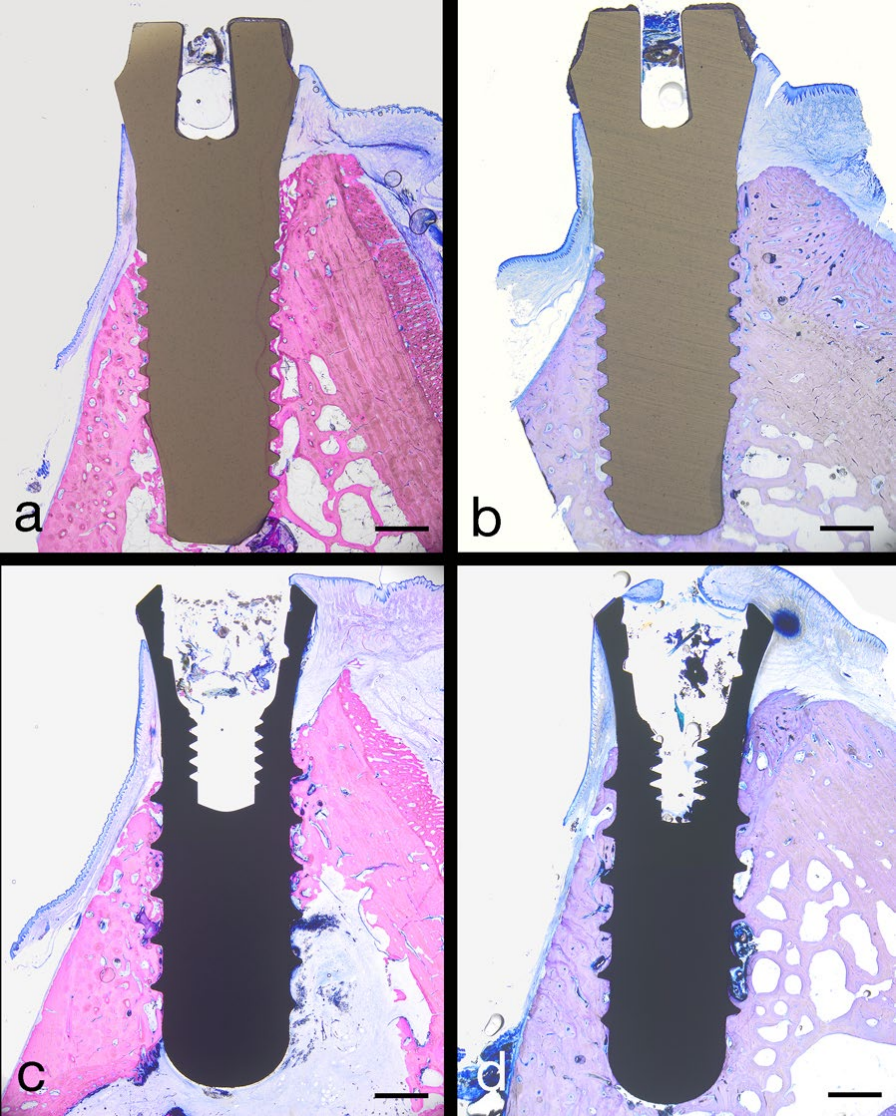
Light microscopic (LM) micrographs showing representative overviews of longitudinal sections through YTZP implants following 4 weeks (**a**) and 8 weeks (**b**). Overviews of Ti-SLA implants are given in (**c**, following 4 weeks) and (d, following 8 weeks). Scale bars: 1 mm

After 8 weeks, nine implants from the test group and two implants from the control group were available for analysis. The histology of one test implant could not be completed because of a failure during the preparation of the section. Figure 3 presents selective light microscopic micrographs of representative specimens after 4 and 8 weeks of transmucosal healing. A high rate of osseointegration, already 4 weeks after implantation, was observed for both types of implants. At 8 weeks, all implants were completely osseointegrated.

The peri-implant alveolar crest was generally characterized by crestal bone resorption. For both implant types, crestal remodeling at the buccal aspect produced dehiscence defects, whereas gap-type bone defects occurred at lingual sites. Crestal bone resorption was measured from the implant shoulder to the most coronal BIC: after 4 weeks a mean bone loss of 5.1 mm (SD ± 0.26) at the lingual sides, and 6.3 mm (SD ± 0.62) at the buccal sides was observed; after 8 weeks a significant higher bone loss of 6.1 mm (SD ± 0.7) was found at the lingual sides (*p* = 0.037), and 6.7 mm (SD ± 0.45) at the buccal sides.

### Histology and histomorphometry of the peri-implant bone

**Fig. 4:**
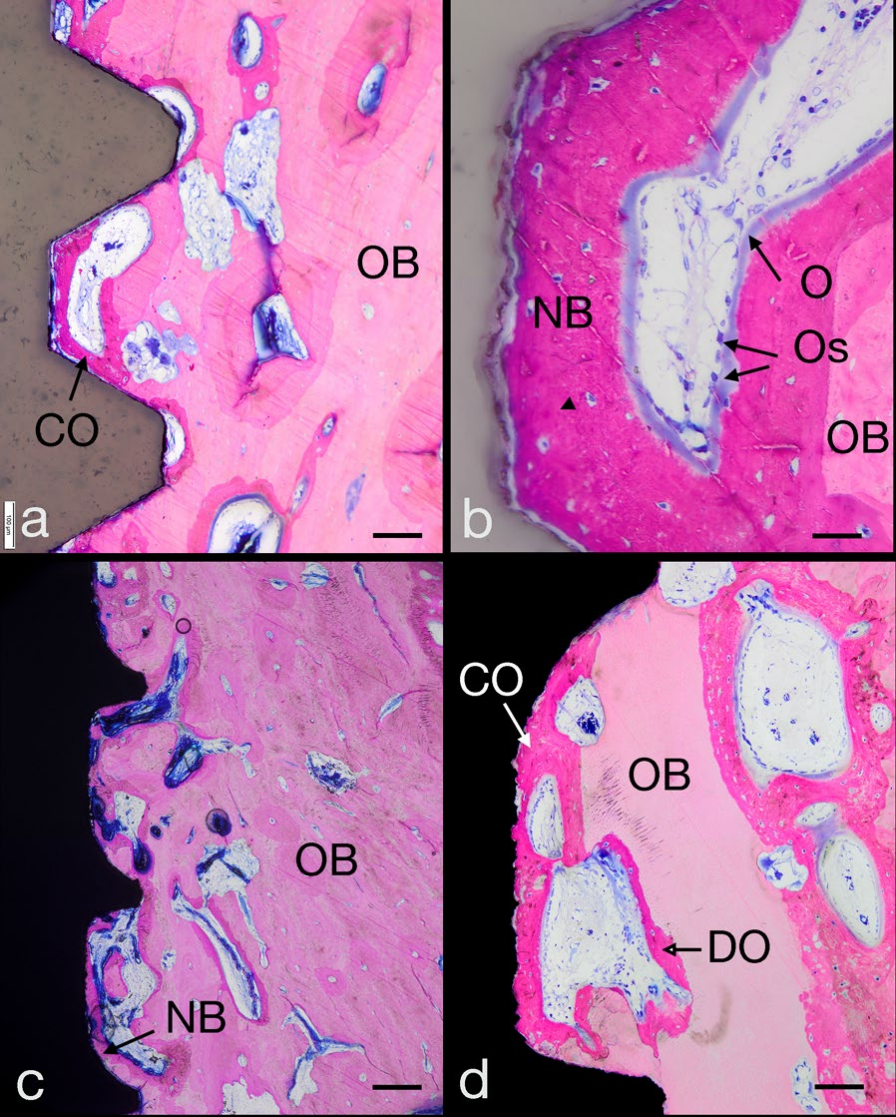
LM micrographs demonstrating the status of osseointegration after 4 weeks with YTZP implants (**a**, **b**) and Ti-SLA implants (**c**, **d**). Note the presence of distance osteogenesis (DO), outgoing from local bone, and contact osteogenesis (CO), outgoing from contact points between local bone with the implant. Also note the presence of an osteoid (O) lined with osteoblasts (OB), indicating ongoing bone formation (**b**, **d**). Scale bars: (**a** and **c**) 100 µm; (**b** and **d**) 300 µm

After 4 weeks of healing, the test and control implants exhibited direct osseous integration and presented a mean BIC of 73.7% (SD ± 16.8) (*n* = 10) for the test implants and 58.5% for the control implant. Distance osteogenesis from local bone towards the implant surface was observed. Moreover, contact osteogenesis starting from contact points between the local bone with the implant was observed directly on and along the surface. Both types of new bone formation were present in the test and control implants (Fig. 4a and c). Ongoing bone formation, indicated by the presence of a not yet mineralized, collagenous osteoid lined by numerous osteoblasts, was evident (Fig. 4b and d).

**Fig. 5:**
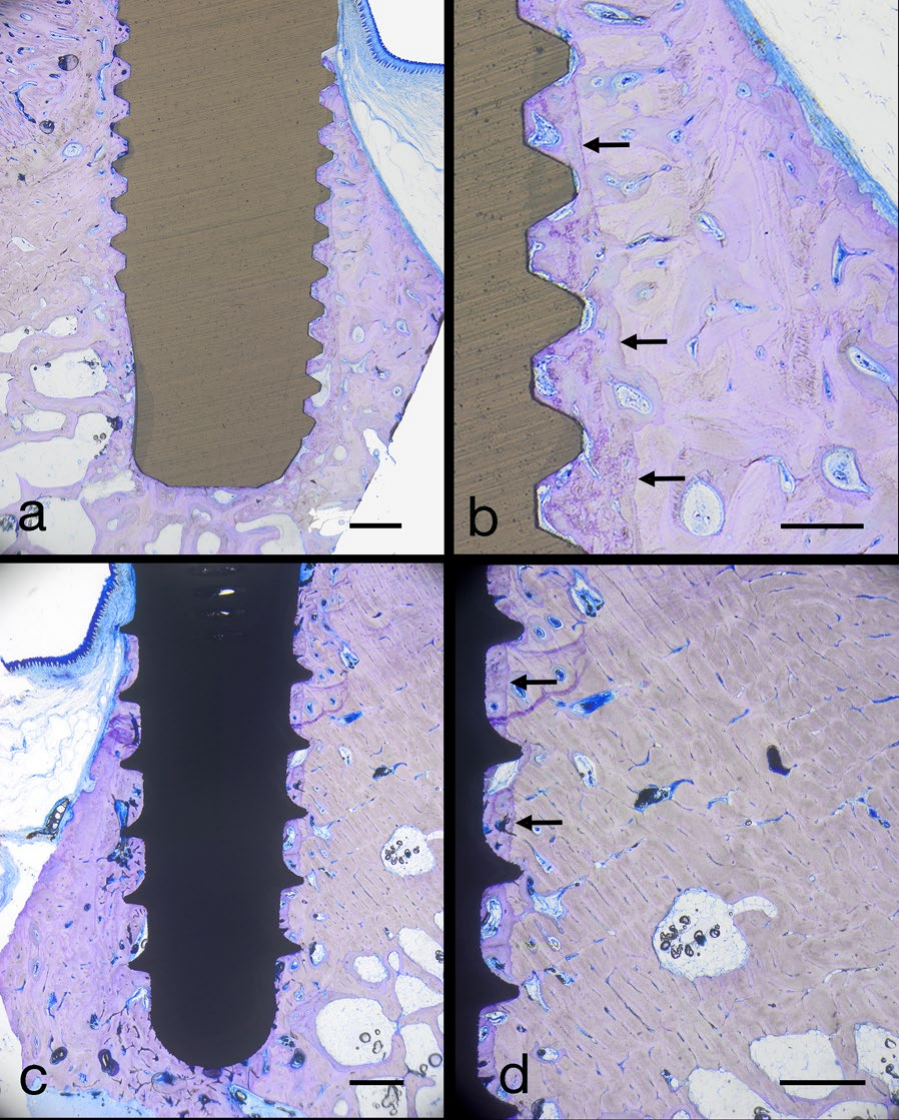
LM micrographs showing complete osseointegration after 8 weeks around YTZP implants (**a**, **b**) and Ti-SLA implants (**c**, **d**). Note the osteotomy walls (black arrows in **b**, and **d**). Scale bars: 1 mm

**Fig. 6:**
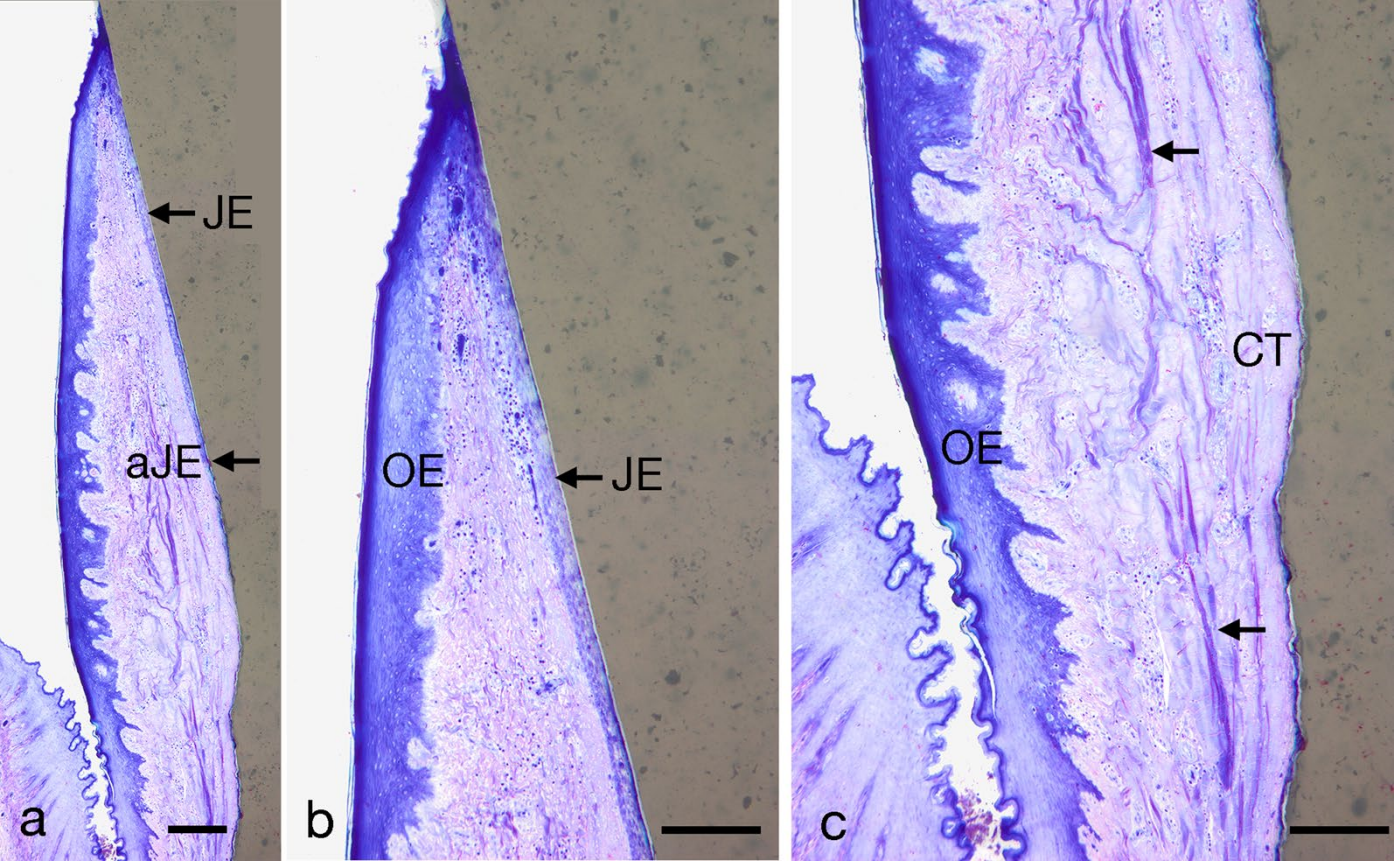
LM micrographs revealing the buccal peri-implant mucosa after 8 weeks. **a** Note the intimately adherent junctional epithelium (JE) and its apical end (aJE). **b** Higher magnification of **a**. Note the keratinized oral epithelium (OE) and the non-keratinized junctional epithelium (JE). **c** Note the collagenous fibers (black arrows) in the peri-implant connective tissue running parallel to the implant surface. Scale bars: 300 µm

Test and control implants were completely osseointegrated after 8 weeks of healing, confirming the null hypothesis. As primary outcome measurement, mean BIC values of 82.4% (SD ± 16.9) for the nine test implants and 93.6% (SD ± 9.1) for the two control implants were observed. No statistical difference was observed comparing 4 and 8 weeks YTZP data (*p* = 0.126). The spaces between the osteotomy walls and the implant surface were filled with newly formed bone and small bone marrow chambers (Figs. 5a–d, 6a, b).

## Discussion

In this study, all evaluated test and control implants were clinically and histologically osseointegrated into our miniature pig model. A well-established method to evaluate the interface and the status between the bone and implant is the measurement of the BIC area by histomorphometric analysis. In the present study, BIC was measured at 4 and 8 weeks. At 4 weeks, the mean BIC for all YTZP test implants (73.7%) was higher compared to the Ti control implant (58.5%). At 8 weeks, the mean BIC ratio was comparable for both materials and in accordance with the criteria defined by Albrektsson et al. [31], indicating successful osseointegration. Other studies comparing zirconia implants to titanium controls confirm our findings: Gahlert et al. [32] report a BIC of 54.6% (ZrO_2_) and 44.1% (Ti), and Linares et al. [33] of 85.4% (ZrO_2_) and 84.3% (Ti), respectively, at 8 weeks.

Several studies describe a stronger bone response for zirconia implants with rough surfaces compared with Zirconia implants with machined surfaces [32, 34, 35]. A study conducted in mini pigs compared machined and sand-blasted zirconia implants and concludes that surface characteristics strongly influence bone integration [34]. In particular, ZrO_2_ implants were biomechanically and histomorphometrically compared with sand-blasted, large grit, acid-etched (SLA) Ti implants. The results indicated higher bone stability values for the SLA Ti implants, followed by the rough zirconia and the machined zirconia surface, suggesting that rough zirconia implants achieve higher anchorage in bone compared with machined zirconia implants.

In a mini pig study conducted by Gahlert et al. [32], the authors compared the bone tissue response of surface-modified Zirconia and Ti implants. For the test group, cylindrical low-pressure injection-molded zirconia implants were created, including an acid-etched surface. SLA Ti implants of identical shape served as controls. A histomorphometric analysis of bone density and BIC ratio revealed no significant differences between the two implant types. The interface between newly formed bone and both the zirconia and Ti surfaces revealed a thin layer of bone following the contour of the threads, thus indicating osteoconductive bone formation.

Another study with mini pigs compared one-piece yttria-stabilized ZrO_2_ implants with a sand-blasted surface (R_a_ = 1.0 µm) to SLA Ti implants (R_a_ = 2.75 µm) [35]. The ZrO_2_ implants were subjected to alternating submerged and non-submerged healing, whereas the Ti implants were all submerged. After a healing period of 4 weeks, both types of submerged implants achieved a BIC ratio of 53%. For the non-submerged implants, some epithelial downgrowth and crestal bone resorption was reported, resulting in a BIC of 48%. The upper-third of the implants exhibited bone formation by contact osteogenesis for both submerged and non-submerged implants. Apically to this zone, distant osteogenesis was observed in all groups.

Cionca and coworkers suggested that the surface on the intraosseous portion of a ZrO_2_ dental implant should be as rough as possible [36]. However, because of the tough material properties of zirconia and depending on the manufacturing process, there is significant variation in the surface roughness between the different manufacturers. The zirconia implant used in the present study, had an especially rough surface, which was approximately five times rougher than other documented ZrO_2_ implants [25, 37]. This could be one explanation for the markedly higher BIC value after 4 weeks of healing compared to other studies in the mini-pig mandible using smoother ZrO_2_ implants [34, 35].

Another experimental study conducted in mini pigs [38] compared ZrO_2_ implants with a modified ablative surface and acid-etched Ti implants following 1, 4, and 12 weeks of healing using scanning electron microscopy. At 1 week, a marked attachment of bone was detected, which was further increased to intimate bone contact after 4 weeks. At 12 weeks, osseointegration was complete. It is unclear whether the tissue observed after 1 week was indeed newly formed bone or the result of the implant insertion procedure. It is known, that during implant placement a rough implant surface acts like micro-grained sandpaper. It scratches along the walls of the cortical and trabecular bone and grinds bone at the interface resulting in several micron-thick smear layers consisting of bone debris and blood, covering part of the implant surface immediately following installation [39, 40, 41]. The presence of bone debris is of crucial importance to speed up the initial bone formation as described by Bosshardt et al. [41]. The bone debris guides new bone formation by distance osteogenesis to the implant surface.

In this study, bone resorption following extraction and implant installation was more pronounced on the buccal as compared to lingual sides. This is well in line with earlier findings reported by Botticelli and coworkers [42].

The results of the present study have to be interpreted cautiously given the small number of test and control implants. In general, a robust statistical analysis of the results could not be performed because of the small sample size. Unfortunately, one control implant was placed in the remnants of a tooth root, which created inflammation and bone resorption and had to be excluded from analysis, limiting the interpretability of the study results. BIC evaluation and interpretability could have been further influenced by the fact, that implant installation causes bone debris and a bone smear layer at the bone–implant interface, as discussed before. The rougher test implant surface (R_a_ = 5.7 µm) may have generated more osteogenic bone debris and a smear layer compared with the control implants (R_a_ = 1.25 µm) and increased the BIC at 4 weeks over proportional. On the other hand, a rougher implant surface may also be a source of enhanced bacterial adhesion and must be taken into account for clinical application [43, 44]. Therefore, larger studies are needed to confirm the present results.

## Conclusion

The results indicate rapid and predictable osseointegration of immediately placed YTZP implants with a rough BBS surface. A high BIC value after 4 weeks of healing was maintained after 8 weeks. The results of this histologic study demonstrate a higher mean BIC on the tested rough zirconia implants compared with previous studies evaluating surface-modified ZrO_2_ implants in similar animal models. The evaluated BBS ZrO_2_ surface may be classified as highly osteoconductive.

## Data Availability

All data and materials are available through Peter Schupbach.

## Abbreviations

BBS: Blasted before sintering
BIC: Bone-to-implant contact
CO: Contact osteogenesis
DO: Distance osteogenesis
JE: Junctional epithelium
O: Osteoid
OB: Osteoblast
SLA: Sand-blasted, large grit, acid-etched
Ti: Titanium
Zirconia: Zirconium dioxide ZrO_2_
ZrO_2_: Zirconium dioxide
YTZP: Yttria-stabilized zirconia
